# Epidemiology and Trend of Head and Neck Cancers in Iran

**DOI:** 10.5539/gjhs.v8n1p189

**Published:** 2015-05-15

**Authors:** Maryam Mirzaei, Seyedeh-Akram Hosseini, Mahshid Ghoncheh, Fahimeh Soheilipour, Shahin Soltani, Fatemeh Soheilipour, Hamid Salehiniya

**Affiliations:** 1 School of Public Health, Tabriz University of Medical Sciences, Tabriz, Iran; 2 School of Nursing (Quchan Branch), Mashhad University of Medical Sciences, Mashhad, Iran; 3 School of Public Health, Hamadan University of Medical Sciences, Hamadan, Iran; 4 Minimally Invasive Surgery Research Center, Iran University of Medical Sciences, Tehran, Iran; 5 School of Public Health, Tehran University of Medical Sciences, Tehran, Iran; 6 Department of Oral Medicine, School of Density. Islamic Azad University, Isfahan (Khorasgan), Iran

**Keywords:** cancer, Iran, head and neck, epidemiology

## Abstract

**Introduction:**

Head and neck cancers are the sixth common cancer worldwide. It is necessary to inform the trend of incidence for health planning. This study aimed to investigate the trend of head and neck cancers in Iran.

**Methods:**

This study was carried out based on national report on cancer registry in Iran. The crude incidence rate was calculated as per 100,000 people, and Age Standardized incidence Rate (ASR) was estimated using direct standardization and the standard population of World Health Organization (W.H.O). Data was analyzed using the Cochran - Armitage test for linear trend and software of WinPepi 2.1.

**Results:**

A total of 25,952 cases of cancers of the head and neck have been registered between 2003 and 2009. The age-standardized incidence rate reached from 4.8 cases per 100,000 in 2003 to 8.5 and 7.4 in 2008 and 2009, respectively, which revealed significantly increasing trends.

**Conclusions:**

According to increasing trend age-standardized rate of head and neck cancer in Iran, it is recommended to identify risk factors and vulnerable groups in order to reduce the burden of this type of cancers.

## 1. Introduction

Cancer is as a major health problem in the world. It is the third leading cause of death in Iran (Naeimeh et al., 2015). Head and neck cancers is defined as malignant tumors of the airways and upper digestive system (Döbróssy, 2005), and known as the sixth common cancer worldwide (Braakhuis, Leemans, & Visser, 2014). The anatomical areas involved the cancers are the oral cavity, nose, nasopharynx, oropharynx, hypopharynx, larynx, thyroid, and salivary glands (Krishnatreya et al., 2014). Head and neck cancers are accounted for the annual incidence of 690,000 cases (4.9% of incidence of all cancers) and almost 375,000 deaths (4.6 of all deaths from cancers) in the world in 2012 (Ferlay et al., 2015). The trend of incidence and mortality of this type of cancers are different according to organ involved, gender, and geographic location (Simard, Torre, & Jemal, 2014).

The incidence of the cancers has reported from different countries in the world about 5 to 50% so that South Asia and parts of southern Europe had the highest incidence (Simard et al., 2014). Although this type of cancers is more common in men, the sex ratio is different by geographical regions and the anatomical areas involved (Blomberg, Nielsen, Munk, & Kjaer, 2011; Braakhuis et al., 2014).

Various genetic and environmental factors, including smoking, alcohol consumption, human papillomavirus, and poor oral hygiene and nutrition, are effective in developing head and neck cancers (Döbróssy, 2005; Karligkiotis et al., 2014; Siegel, Naishadham, & Jemal, 2013). Of the above risk factors, smoking and alcohol consumption are the main risk factors for the cancers (Simard et al., 2014). Over the past two decades, since the prevalence of smoking has reduced in many developed countries, recently, the role of human papillomavirus in developing this type of cancers has been more prominent (Blomberg et al., 2011; Kreimer, Clifford, Boyle, & Franceschi, 2005).

To compare the incidence of any disease in different populations, use of the Age Standardized incidence Rate (ASR) is important indicator (Mousavi et al., 2010).

The highest ASR of head and neck cancers has been reported from countries like France, Brazil, India, and Black America (Elango, Gangadharan, Sumithra, & Kuriakose, 2006). Approximately 36,500 new cases and 11,000 deaths from head and neck cancers occur in United States (Siegel et al., 2013). In Australia, the cancers with the incidence of 12.3 per 100,000 of are the fifth common cancer (Jayaraj, Singh, Baxi, Ramamoorthi, & Thomas, 2014).

Considering different epidemiology of the cancers in various countries, and lack of information on the incidence and epidemiology in the whole of Iran, the present study aimed to investigate the trend of head and neck cancers in Iran.

## 2. Methods

### 2.1 Data Source and Study Population

This study was carried out based on existing data, obtained from the national report on cancer registry and disease management center of ministry of health in Iran (Goya, 2007). The existing data contained data from 41 pathology centers of medical university in the country between 2003 and 2009. All registered cases were studied by each province. The incidence rate was calculated as per 100,000 people and ASR using direct standardization and the standard population of World Health Organization (W.H.O). The data collected is encoding using ICD – O, which related to the code C00-06, 07-08, 09-13, 32 and 73.

### 2.2 Statistical Analysis

The number of cases, and crude and standardized incidence rates was also examined by sex and each province. Data was analyzed using Cochran - Armitage test for linear trend and software of WinPepi 2.1.

## 3. Results

In this study, all cases of head and neck cancers were examined based on cancer registry data. The frequency distribution of cases, crude and standardized incidence rates are shown by age group, sex in Table 1. A total of 25,952 cases of cancers of the head and neck have been registered between 2003 and 2009. Of these cases, 14,021 cases (54%) were men and 11 931 cases (46%) women. Sex ratio (male to female) was greater than one for various types of head and neck cancers, except for thyroid cancer, in all the years studied. According to Table 1, crude and age-standardized incidence rate per 100,000 were more in men than women for different types of head and neck cancers, except for thyroid cancer, and have increased over time. The highest incidence rate was seen in the age group of 80 to 84 years in both sexes.

**Table 1:** Numbers of incident cases of head-and-neck cancer (HNC) and distribution by site and gender, IRAN, 1382-1388

Site of HNC	Sex	N(%)	CIR	ASR	M/F
Lip & oral cavity	Male	2775(55.7)	7.72	9.7	1.25
	Female	2218(44.4)	6.55	9	
	Total	4993	7.2	9.3	
Parotid & salivary gland	Male	916(57.4)	2.54	3.2	1.34
	Female	680(42.6)	2	2.5	
	Total	1596	2.3	2.6	
Pharynx & Tonsil	Male	1935(62.9)	5.36	6.7	1.79
	Female	1080(35.1)	3.31	4.19	
	Total	3075	4.1	5.5	
Larynx	Male	5798(88.4)	16.07	21.6	7.62
	Female	760(11.6)	2.31	3.2	
	Total	6558	9.1	12.4	
Thyroid	Male	2597(26.5)	7.17	8.3	0.36
	Female	7193(73.5)	21.21	24.1	
	Total	9790	14.1	16.3	
All	Male	14021(54)	38.2	45.9	1.17
	Female	11931(46)	35.4	43	
	Total	25952	36.8	46.3	

**Figure 1:**
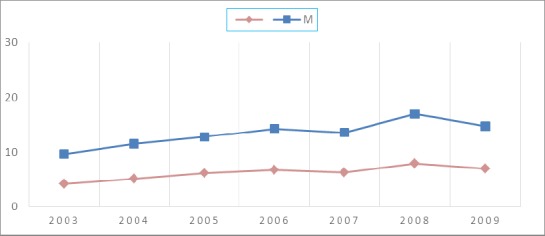
Trend of HNC in the IRAN by Sex, Over 7 Year

The age-standardized incidence rate per 100,000 is shown in Figure 1 during 2003 to 2009. As can be seen, the head and neck cancers had a significant increased trend. There was an increased of incidence in both sexes so that in 2003 the incidence of head and neck cancers in both men and women was 4.2 and 5.4, respectively. However, the incidence reached from 9.1 in 2008 to 7.8 in 2009 in men and 7.9 in 2008 to 6.9 in 2009 in women. The Cochran - Armitage test indicated that a significant trend in the incidence rate of the cancers (Chi^2^= 559.85, P = < 0.001).

## 4. Discussion

Our findings showed that the age-standardized incidence rates of the head and neck cancers were 4.8, 5.8, 6.4, 7.2, 6.8, 8.5 and 7.4 cases per 100,000 during 2003 to 2009 in Iran. There was an increasing and significant trend (P = < 0.001). However a slight decrease was seen in the age-standardized incidence rates of the cancers in 2009 compared with 2008, the rate increased 1.5 twice during years of the study. This may be due to more comprehensive implementation of cancer registry programs in some years studied.

In our study, ASR for all types of head and neck cancers was 46.3 cases per 100,000 people (in men and women 49.5 and 43, respectively. This indicated that a high level of this type of cancers in the country, similar to other studies in the worlds (Jayaraj et al., 2014). A study performed in Denmark showed a significant increase per year for the cancers and the highest age-specific incidence in individuals older than 60 years (Blomberg et al., 2011).

A study on the trend of incidence of the cancers between 2007 and 2010 in Australia revealed that although the incidence of head and neck cancers were declining in men and in women were fixed, the overall incidence of head and neck cancers was high (21 per 100,000) (Jayaraj et al., 2014).

The trend of incidence of the cancers is different by the organ involved, gender, and geographic location (Simard et al., 2014). The trend of cancer of the oral cavity is increasing in both sexes in some Asian and European countries such as Japan and Finland, and only in women in France and Italy. In a study conducted in India, this trend was different in various regions and both sexes (Yeole, 2007).

Findings obtained from a study in an area of Italy showed increasing trend in the incidence of this type of cancers for women and a slight decrease for men, and sex ratio (male to female) of 5.1 and the mean age of 62 years (Karligkiotis et al., 2014). Laryngeal cancer is the most common type of head and neck cancers in both sexes in Iran (Mafi, Kadivar, Hosseini, Ahmadi, & Zare-Mirzaie, 2012).

Like many other studies, the sex ratio (male to female) was larger than one in our study, except thyroid cancer (Blomberg et al., 2011; Elango et al., 2006; Jayaraj et al., 2014; Mishra & Meherotra, 2014). Greater incidence of head and neck cancer in men can be due to higher proportion of smokers among men than women (Larizadeh, Damghani, & Shabani, 2014). However, the sex ratio is different depending on the anatomical involvement (Mehanna, Paleri, West, & Nutting, 2010).

In our study, 54% of males and older age groups in both sexes had the highest incidence rate. The results of various studies on the general characteristics (age and gender) of this type of cancers were also similar. Overall, the difference in the increase or decrease trend of this type of cancers can be caused by differences in exposure to various environmental risk factors.

## 5. Conclusion

According to increasing trend age-standardized rate of head and neck cancer in Iran, it is recommended to identify risk factors and vulnerable groups in order to reduce the burden of this type of cancers.
